# Implementation of High-Performance Blockchain Network Based on Cross-Chain Technology for IoT Applications

**DOI:** 10.3390/s20113268

**Published:** 2020-06-08

**Authors:** Ting Lin, Xu Yang, Taoyi Wang, Tu Peng, Feng Xu, Shengxiong Lao, Siyuan Ma, Hanfeng Wang, Wenjiang Hao

**Affiliations:** School of Computer Science and Technology, Beijing Institute of Technology, Beijing 100081, China; linting@bit.edu.cn (T.L.); 2220170695@bit.edu.cn (T.W.); pengtu@bit.edu.cn (T.P.); 3220190986@bit.edu.cn (F.X.); 3220190951@bit.edu.cn (S.L.); 3220190968@bit.edu.cn (S.M.); 3220190980@bit.edu.cn (H.W.); 3220190943@bit.edu.cn (W.H.)

**Keywords:** IoT, blockchain, high-performance, consensus algorithm, cross-chain protocol

## Abstract

With the development of technology, the network structure has changed a lot. Many people regard the Internet of Things as the next-generation network structure, which means all the embedded devices can communicate with each other directly. However, some problems remain in IoT before it can be applied in a large scale. Blockchain, which has become a hot research topic in recent years, may be one of the solutions. However, currently, the transaction speed of blockchain is still a disadvantage compared to traditional transaction methods. This paper focuses on to implement a high-performance blockchain platform. After investigation of the current blockchain consensus algorithm and blockchain architecture, we propose: (1) an improved blockchain consensus algorithm, which is implemented based on the mortgage model instead of probability model; (2) a cross-chain protocol with transverse expansion capacity, which would support the message transmission among chains; (3) a high-performance cross-chain blockchain network structure, which could handle more than 1000 transactions per second per chain by verification. Experiments have been carried out, and shown that the cross-chain blockchain network structure we provided is feasible to meet the requirement of large-scale distributed IoT applications.

## 1. Introduction

With the development of technology, the network structure has changed a lot. Many people regard the Internet of Things as the next-generation network structure, which means all the embedded devices can communicate with each other directly. But some problems remain in IoT before it can be applied in a large scale. Blockchain, which has become a hot research topic in recent years, may be one of the solutions [1,2,3,4,5].

Blockchain technology could provide a way to track the unique history of individual devices in the IoT network by recording a ledger of data exchanges between it and other devices, web services, and human users. The tokenization feature of a blockchain IoT also means that smart machines can be used for revenue generation. Through tokenization, IoT device owners will be able to use blockchain to manage and sell data they generate for digital currency. In summary, the potential for Blockchain and IoT is just beginning to become apparent as many companies explore use-cases for business optimization.

Bitcoin [6] was born in 2009, as an electronic cash currency [7] has become a social phenomenon-level topic, pioneered the decentralized encryption currency [8], and gave birth to the hot research of its underlying technology blockchain [9].

Ethereum [10], known as blockchain 2.0, is not only limited to the field of digital money, but also characterized by “programmable blockchain”, which makes it a platform for decentralized application development, and has a profound impact. Zcash [11] and Monero [12], known for their anonymity, have also been successful. In the ten years since Bitcoin was born, many blockchain projects have been developed and have achieved varying degrees of success.

However, most blockchain projects still face many challenges due to the limitations of the blockchain technology architecture, such as the extendability and scalability of the block capacity, the lack of chain governance, the performance bottleneck of the distributed consensus algorithm [13], and the like.

At present, theoretically, the maximum number of transactions that Bitcoin can handle is 7 per second, and the maximum number of transactions can be handled by Ethereum is 20 per second, while the traditional centralized financial system PayPal can handle an average of 193 transactions per second, and Visa can handle 1667 transactions per second [14].

It can be seen that the existing blockchain systems are generally faced with a problem: the limited number of transactions can be processed per second, or we can say the transaction efficiency is too low. So, they cannot meet the needs of large distributed applications. Thus, it is of great theoretical and practical significance to improve the transaction speed of blockchain.

Currently, mainstream blockchain systems, such as Bitcoin or Ethereum, have their own solutions for the purpose of enhancing transaction efficiency. Famous methods for enhancing transaction efficiency for Bitcoin include increase block size [15], SegWit [16], and Lighting Network [17]. As for Ethereum, state-of-the-art methods are Sharding [18], Raiden Network [19], and Plasma [20].

These methods, however, have certain limitations and have no significant effect for the time being. We have analyzed the causes of these solutions and found that there are two main reasons to limit the effectiveness of these solutions: first, these methods are limited to the POW algorithm which is based on the probability model in the choice of the underlying consensus algorithm; secondly, these solutions all used the single-chain architectures, not attempted to adopt the multi-chain architecture. Thus, the effectiveness of optimization on the transaction efficiency is limited.

In view of this, this paper proposes:

1. An improved blockchain consensus algorithm, which is implemented based on the mortgage model instead of probability model;
2. A cross-chain protocol with transverse expansion capacity, which would support the message transmission among chains;
3. A high-performance cross-chain blockchain network structure, which could handle more than 1 thousand transactions per second per chain by verification.

The following is organized as: Literature concerning this topic will be discussed in Section 2 reviews the literature work; Section 3 introduces the details of the consensus algorithm in this paper; Section 4 presents the cross-chain architecture; while Section 5 gives through discussion of the cross-chain network architecture designed in this paper; Results are shown in Section 6; Conclusion is in Section 7.

## 2. Literature Review

IoT and blockchain are gaining more and more attention due to their success in versatile applications. Khan et al. [2] have taken the secure IoT-blockchain data of Industry 4.0 in the food sector as a research object. They have proposed a hybrid model based on recurrent neural networks (RNN). Jamil et al. [5] proposed a novel platform for monitoring patient vital signs using smart contracts based on blockchain. The proposed system is designed and developed using hyperledger fabric, which is an enterprise-distributed ledger framework for developing blockchain-based applications. Ali et al. [21] presented a solution for patients to share their biomedical data with their doctors without their data being handled by trusted third-party entities. The solution is built on the Ethereum blockchain as a medium for negotiating and record-keeping, along with Tor for delivering data from patients to doctors. Rathee et al. [22] have addressed the issue of security in smart sensors of connected vehicles that can be compromised by expert intruders through a blockchain framework. This study has further identified and validated the proposed mechanism based on various security criteria, such as fake requests of the user, compromise of smart devices, probabilistic authentication scenarios and alteration in stored user’s ratings. Jo et al. [23] have combined IoT with blockchain-based smart contracts for structural health monitoring of underground structures to define a novel, efficient, scalable, and secure distributed network for enhancing operational safety. In this blockchain IoT network, the characteristics of locally centralized and globally decentralized distribution have been activated by dividing them into core and edge networks.

However, as we discussed before, the transaction efficiency has become a problem restricting the application of blockchain for large distributed business applications. Many works concerning this issue have been reported in the literature.

In history, Bitcoin has repeatedly increased the size of the block to increase the speed of the transaction [15]. In 2010, Satoshi Nakamoto proposed a 1 MB block cap to withstand Dusting Attack. In 2015, Gavin Andreesen proposed a 20 MB block cap. However, this approach not only increased customs’ cost, and also had a very limited increase on the transaction efficiency. Moreover, larger blocks will lead to higher operating costs for the whole node, fewer individuals with complete nodes, and higher centralized hash power [24].

In 2015, Pieter Wiulle proposed SegWit [16]. SegWit recommends storing digital signatures in separate blocks, freeing up space and causing more transactions per block. It divides the transaction into two parts, removes the digital signature from the original part, and appends it as a separate structure at the end [25]. But opponents argue that the complexity of updating is one of the main problems. Most importantly, special nodes are needed to store signature data, which is contrary to the idea of distributed network conceived by Satoshi. In any case, after SegWit proposed, the number of transactions Bitcoin can handle has doubled in two days.

In 2015, Joseph Poon and Thaddeus Dryja have presented Lightning Network [17]. It is a two-layer payment protocol that runs on blockchains. It can theoretically implement fast transactions between participating nodes and is touted as a solution to Bitcoin scalability. The normal use of lightning network includes opening payment channels by submitting financing transactions to the relevant blockchains, then carrying out any number of lightning transactions, updating the provisional allocation of channel funds without broadcasting to the block chain, and selectively closing the payment channels to allocate channel funds through the final version of the broadcast transactions. But the introduction of lightning networks may also have the following shortcomings: lower costs may undermine the availability of Bitcoin; because of the broadcast effect of lightning networks, users will not see “every” transaction, thus reducing transparency.

The Sharding [18] scheme for Ethereum was inspired by traditional database. It attempts to distribute the independent data in the block to each node in the block chain, which not only reduces the amount of data stored by each node, but also improves the speed of node verification. However, there are still many problems to be solved to fully implement it, such as the problem of “no proof” [26]. Sharding technology can expand to some extent, but putting all transactions on the block chain is not a long-term solution. Since 2017, the Ethereum blockchain has expanded rapidly at a rate of about 2.5 GB per day, and the volume has grown by nearly 100 GB in just one month. Such a large volume will increase the operating cost of the node and reduce the synchronization efficiency of the blockchain. Therefore, in addition to the in-chain expansion scheme, there is also a need for out-of-chain expansion scheme.

Similar to Lightning Network, instead of putting all the transaction information on the block chain, Raiden Network [19] scheme try to transfer some transactions outside the chain, allowing users to exchange transfer signature information in private to achieve the transaction. The difference is that Raiden Network in theory can handle “status transactions”, i.e., non-monetary transactions with similar concepts, on the basis of completing the payment task under the chain.

In 2017, Vitalik and Joseph Poon presented a scheme called Plasma [20]. In this project, they design the blockchain as a tree structure. The concept of “sub-blockchain” is proposed to reduce the amount of data stored on the blockchain, and a technique called “fraud proof” is used to connect the “sub-blockchain” and the “main blockchain” in order to improve the transaction processing ability of Ethereum.

Increasing the block size only increases the number of transactions that the block can accommodate, but does not change the consensus algorithm that affects the consensus reached by the node, thus the effect of increasing transaction efficiency is not significant. The SegWit scheme introduces a new DOS attack vulnerability. When an attacker tries to construct a huge block with a large number of transactions, if other nodes in the network try to verify the block, it is necessary to obtain the witness data from the attacker, which leads to a sharp increase in the verification time of the block and the blockage of the node. Although lightning network can meet the demand of timely micro-payment to a certain extent, it is not suitable for large payment and offline trading, and there are hidden concerns about security problems. As for schemes proposed for Ethereum, although some of them are practical in theory, many issues remain unsettled in the time being.

According to the literature review, this paper proposes to enhance the transaction efficiency of blockchain through: (1) optimization of consensus algorithm of nodes; (2) expand from single-chain architecture to multiple-chain architecture.

Many researchers have contributed on related subjects recently. Wang et al. [27] have investigated the cross-chain communication, and introduced blockchain router, which empowers blockchains to connect and communicate cross chains. However, the blockchain router can only act as a router, and the chains in this system cannot communicate messages between other chains directly, while in our work, the chain designed can communicate messages between each other directly. Boyen et al. [28] have proposed a blockchain-free cryptocurrency, which they introduced as a framework for truly decentralized fast transactions. The distributed ledger system in their work is based on a lean graph of cross-verifying transactions, and the distributed consensus mechanism is based on progressive proofs of work with predictable incentives. Martino et al. [29] introduced Chainweb, which they named a proof-of-work parallel-chain architecture for massive throughput. Their work mainly focusses on improving the performance of POW by combining hundreds or thousands of individually mined peer chains into a single network, which is different from our blockchain architecture. Herlihy [30] proposed an atomic cross-chain swap protocol, using a form of hashed timelock contracts. This paper mainly focusses on atomic cross-chain swaps, while ours is not based on atomic cross-chain swaps to achieve the cross-chain results.

In this paper, a cross-chain protocol is designed so that the two blockchains can communicate with each other and transmit transaction messages while dealing with each other. The consensus algorithm is improved, the POW [31] algorithm based on probabilistic model is abandoned, and the POS [32] algorithm based on entrusted mortgage model is adopted. The accounting right is limited to the outgoing block node, to shorten the consensus time of the node and improve the transaction processing efficiency of the chain.

## 3. Improvement of Consensus Algorithm

### 3.1. Overview

The essence of blockchain is a public ledger, in which the whole process of the state transition of the state copy machine is recorded. Each node in the chain has the right to copy this public ledger. In Bitcoin and Ethereum, since probabilistic model-based Proof-Of-Work (POW) algorithm is used at the node consensus level, any node in the network has the opportunity to write new record to this public ledger.

In this section, we will present our improvement on the consensus algorithm. We have a way to effectively solve Byzantine problem at the consensus level. If there is fraud in the chain, it is always possible to verify who is the perpetrator. The improved consensus algorithm in this paper is not only suitable for the cross-chain network designed by us, but also suitable for the single-chain blockchain architecture.

The algorithm designed in this paper divides the nodes in the blockchain into ordinary nodes and producer nodes. The ordinary nodes are responsible for forwarding and relaying the transactions in the chain, while only producer nodes have the right to write new record (transactions) to the blockchain’s public ledger.

Producer nodes are identified by their public key. Producer nodes are responsible for maintaining a full copy of the state copy machine, proposing new blocks and voting for proposed new blocks. The producer node will assign incremental height level to each new added block so that the blockchain has one and only one valid block at each height level. At each block height level, producer nodes will take turns to propose new block, in a way that, for any given round, there is at most one valid proposer.

Due to the asynchronous nature of the network, multiple rounds may be required to submit a new block at a given height level. If more than one-third producer nodes are in offline or network-partitioned status, the blockchain network may stop. The producer nodes will perform two stages of vote before submit a new block, with help of a simple locking mechanism, which could prevent a malicious alliance of less than one third of the producer nodes from compromising the security of the entire chain.

Each block will contain some metadata in its block header including a hash of the block of the previous height level to produce a hash chain. The block header also contains the height level of the block, the local time of the proposed block, and the Merkle root hash of the transaction contained in the block.

### 3.2. Framework of Our Consensus Algorithm

The core part of the consensus algorithm designed in this paper consists of:

1. Proposal mechanism: In each round, only one producer node has the right to propose a new block and broadcast it to other producer nodes. If no proposal of new block is received in time, the proposer will be skipped;
2. Vote mechanism: A two-stage vote must be held to ensure the best Byzantine fault tolerance. These two stages are known as the nominate phase and the pre-submission phase, respectively. If in a round, there are more than 2/3 producer nodes vote for a pre-submission of a block, this will guarantee a submit for that block;
3. Locking mechanism: We rely on the designed locking mechanism to ensure that none two producer nodes could submit different blocks at the same height level, under the assumption that no more than 1/3 producer nodes are malicious.

According to our consensus algorithm, in order to provide tolerance for a single Byzantine fault, the network of the chain must contain at least four producer nodes. Each producer node must have an asymmetric encryption key pair used to generate a digital signature. All producer node starts with same initial state, which includes a list *L*, an ordered list of producer nodes. Each producer node is identified by its public key. All proposals and votes must be signed by the corresponding private key. This ensures that any ordinary node can always verify proposals and votes. If up to 1/3 of the outgoing nodes are malicious, the network stops working, which is helpful to prevent cooperation in any way to undermine the security or activity of the system.

The consensus begins in round 0. The first proposer is the first producer node on list *L*. The results of one round are either submitting a new block or deciding to move on to the next round. Then another producer node could propose. In the case of asynchronous network or producer node failure, the use of multiple rounds mechanism could give producer nodes more chances to reach a consensus. Compared with the algorithm that needs a form of leader election, the consensus algorithm designed in this paper will have a new leader (proposer) in each round. The producer nodes vote to decide whether to transfer to the next round, in the same way as they vote to decide whether accept the proposal, i.e., providing a unified mechanism for the protocol, which does not exist in the algorithm with a clear leader election plan.

The dependence on synchronization at the beginning of each round is weak because the local clock is used to determine when to skip the current proposer. The algorithm sets a TimeoutPropose variable locally in the producer node. If the producer node does not receive a proposal to enter a new round in the locally measured TimeoutPropose, it can vote to skip the current proposer. The mechanism is inherently synchronous, i.e., we assume the proposal should be received within the TimeoutPropose, and the TimeoutPropose itself can be incremented with each round.

**Figure 1:**
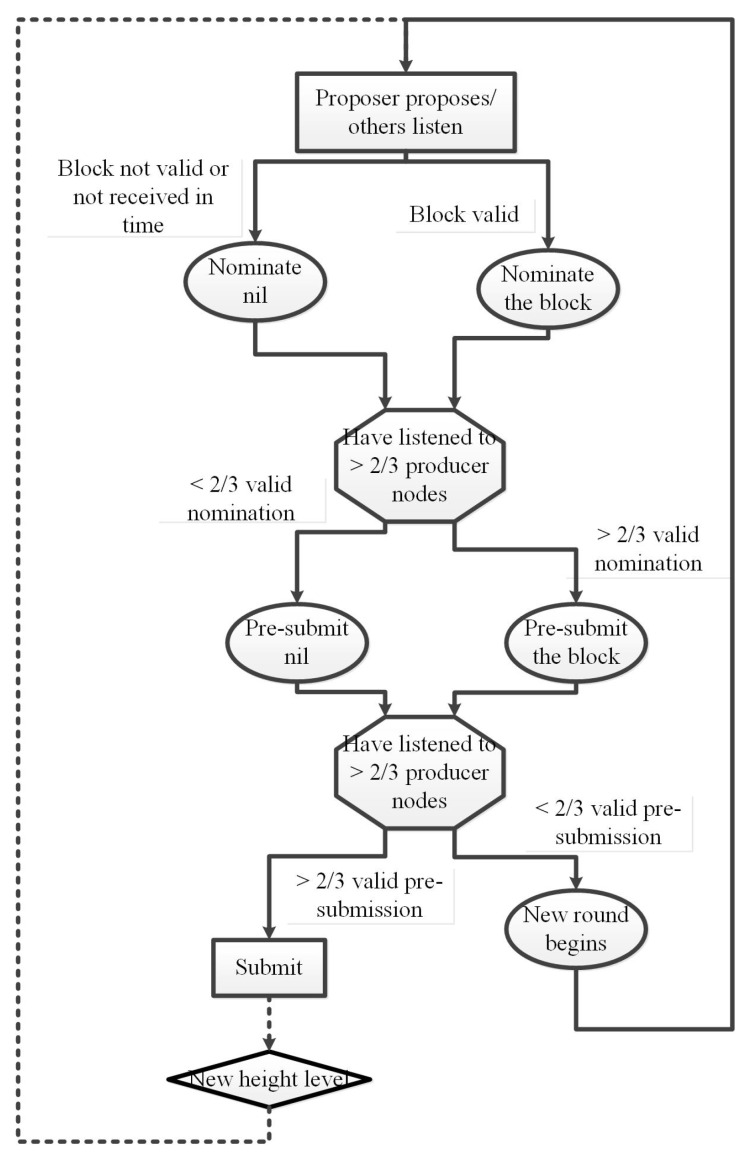
Flow of the designed consensus algorithm.

After the proposal, a round was carried out in a fully asynchronous manner. The producer node will perform next move only when it has listened to at least two-thirds of the other producer nodes. This reduces any dependency on the synchronization clock or bounded network delay, but implies that the network will stop if one third or more producer nodes are not responding. This whole process is shown in Figure 1.

### 3.3. Vote Mechanism

For the sake of security, locking mechanism is introduced in the algorithm, in order to force the producer nodes to prove the validity of their votes. Although the algorithm do not require producer nodes to broadcast their augment data in real time, it do require the producer nodes store their augment data so as to present as evidence when the security issue arose with enough Byzantine faults. This mechanism ensures that the algorithm in this paper has a better chance when facing this kind of failure.

Producer nodes use different kinds of message to communicate, in order to manage the blockchain, the state of application programs, point-to-point network and consensus process.

During the consensus process, only two kinds of message would be used:

1. ProposalMsg: Propose message signed by the proposer at a given height level in a particular round;
2. VoteMsg: Vote message for a proposal.

### 3.4. Proposal Mechanism

Every round begins with a proposal. The proposer of a given round will obtain a batch of recently received transactions from its local transaction memory pool to form a block and broadcast a message which contains that block’s signed proposal. If the proposer of this round is a Byzantine node, it might broadcast different proposal messages to different producer nodes.

A simple deterministic cycle method is used to decide the order of proposers. Only one proposer is valid for a given round, and each producer node knows the correct proposer in any round. If a lower round proposal is received, or if it comes from an incorrect proposer, the proposal will be rejected.

The rotation of producer nodes as proposer is necessary to improve Byzantine fault tolerance. The blockchain designed in this paper solves the security problem through vote and locking mechanism, and keeps the vitality through rotation of the producer nodes.

Once the producer node receives a valid proposal, it nominates the proposal and broadcasts it on the blockchain network. If the producer node does not receive a correct proposal within the time of the ProposalTimeout setting, it will nominate nil.

In an asynchronous environment with Byzantine verifier, a single vote phase (only one vote per verifier) is not enough to ensure security. In essence, because the producer node can be fraudulent, and because there is no guarantee of message delivery time, rogue producer nodes can coordinate some producer nodes to propose, while other producer nodes, because they do not see this proposal, will enter a new round, in which they propose different proposals.

Single-stage vote allows producer nodes to tell each other what they know about the proposal. But to tolerate Byzantine errors, they must also tell each other how much they claim to know about the proposal. In other words, the second vote stage ensures that there are enough producer nodes to witness the results of the first stage.

Therefore, if a producer node vote for a block, it will prepare to submit the block, and if a producer node vote for nil, it will prepare to enter next round. In an ideal situation, more than 2/3 of the producer nodes will vote on the proposal. If in a vote stage, there are more than 2/3 producer nodes give valid vote, we call it a successful vote.

When a producer node receives a successful nominate vote, it will vote for pre-submission of the block. Sometimes, due to network partition, the producer node might not receive a successful vote, or there is no successful vote in the network. In this case, it is unreasonable for the producer node to sign the pre-submission of the block, instead it will sign and publish the nil pre-submission vote. In other words, signing a pre-submission without a successful nominate vote statement would be considered malicious.

The pre-submission of a block is a vote indicating the submission of the block. The pre-submission of nil is a vote indicating the actual need to move on to the next round. When a producer node receives a successful pre-submission vote, it will prepare to submit the block, calculate the result status, and move to round 0 at the next height level. If the producer node receives more than 2/3 of the pre-submission of nil, it will proceed to the next round.

### 3.5. Locking Mechanism

Ensure the security across different rounds can be a thorny issue, because this must be avoided, otherwise it will provide a basis for the submission of two different blocks from two different rounds at the same height level. In the consensus algorithm designed in this paper; this problem is solved by the locking mechanism.

We have defined two rules in the locking mechanism. And we will explain the design motivation by going through two practical scenarios.

**Scenario 1**: Considering a network where there are *A*, *B*, *C*, and *D* four producer nodes. Assume at round *R*, there was a proposal of block *X*, and suppose after the nominate vote stage, block *X* actually received more than 2/3 producer nodes’ nominate votes, which was a successful nominate vote indeed. However, *A* has not seen it, and has pre-submit nil, while other three producer nodes have pre-submitted *X*. Now, assume the only producer node has seen all pre-submission results is *D*, while *B* and *C* have only seen their own pre-submission results of *X* and *A*’s pre-submission result of nil. Thus, *D* would submit *X*, while other three producer nodes would enter round R+1. Since any producer node could be the new producer in round R+1, they might propose block *Y*, and vote for it, even submit *Y*, which would bring harm to the network, since *X* was already submitted. Here, non-Byzantine behavior involved, just caused by network issue.

**Rule 1**: Pre-submit locking: Producer node is considered to be locked to its last pre-submitted block. In a round, a producer is proposer, it proposes, otherwise, it could only vote for the block it locked to. This could prevent that a producer node pre-submit a block in one round, and provide successful vote for another block in next round, thus to enhance the safety of the chain.

Rule 1 is designed to force producer nodes insist on the block they have pre-submitted. In essence, if more than 2/3 producer nodes have pre-submitted for a block, the network might be locked to that block temporarily, which ensures that at the same height level, in a higher round, successful vote for another block is impossible.

**Scenario 2**: Considering a network where there are only *A*, *B*, *C*, and *D* four producer nodes. Suppose in round *R*, *A* and *B* have pre-submitted block *X*, while *C* and *D* have pre-submitted nil. No successful vote, so they all entered round R+1. In round R+1, assume either *C* or *D* has proposed block *Y*, and they have all nominate for *Y*. Suppose *A* is a Byzantine node, so *A* nominate for *Y* (even *A* is already locked to *X*, according to Rule 1). Then, *Y* actually received a successful nominate vote. Assume *A* is offline, while *B* can only vote for *X* according to Rule 1, and *C* and *D* have pre-submitted *Y*. Now, they (*B*, *C*, and *D*) have no possibility to generate a successful vote. In this scenario, less than 1/3 (only one node in this scenario) Byzantine node have ruined the security.

**Rule 2**: Successful vote unlocking: Producer nodes can only unlock when they see a successful vote with round higher than the round it locked to. This allows the producer nodes to unlock when they pre-submit contents that other nodes of the blockchain network do not want to submit, thus maintaining the activity level of the chain, but executing in a manner that does not affect the security.

Locking is not enough, we need unlocking. Under Scenario 2, if *B* has seen the successful nominate vote of *Y* in round R+1, it could unlock, and could pre-submit *Y*. Issue solved.

## 4. Designing of Cross-Chain Architecture

At present, the mainstream blockchain is basically a single-chain structure, i.e., all nodes try to reach a consensus of state and data in the same chain, which undoubtedly limits the performance enhancement effect of the blockchain.

In this paper, we improve this and transform the single-chain architecture into multi-chain architecture. The basic design idea of multi-chain architecture adopts the way of light client and relay node working together. On the premise of ensuring the security of the transaction, the cross-chain transaction is verified by light client, and the relay node is used to broadcast the transaction of the two chains.

### 4.1. Light Client

**Figure 2:**
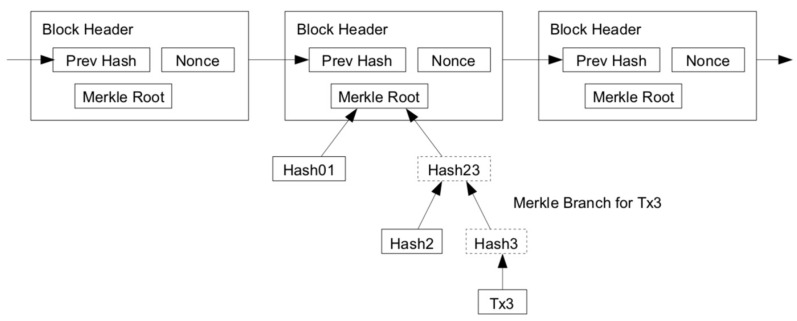
Idea of light client.

The nodes in the blockchain can verify a transaction without running all the nodes of the complete network. The user only needs to keep a copy of the block head of the longest chain (available by querying the nodes in the blockchain network) until he is convinced that he already has the longest chain and gets the Merkle branch [26] that links the transaction to the block. He cannot check the transaction for himself, but by linking it to a place in the chain, he can see that the nodes in the blockchain network have already accepted it and add the new block after further confirming that the network has indeed accepted it (as shown in Figure 2). Therefore, as long as the honest node control network, verification is reliable.

### 4.2. Protocol of Cross-Chain

Cross-Chain protocol is designed based on Cross-Chain relay nodes. Relay is a more “direct” method, which is used to promote the interaction between chains, rather than relying on a third-party trusted intermediary to provide information from one chain to another. The chain itself effectively undertakes the task of broadcasting and verifying cross-chain transactions.

Consider the following scenario: Assume a transaction carried on Chain *B* wants to acquire a specific event on Chain *A*, or the values included in a specific object at a specific time of the status of Chain *A*. And assume Chain *A* is designed with a style alike Bitcoin or Ethereum, where there also are the concepts of “block” and “block head”, while “block head” here represents whole information data (might also be status data) of the ”block”.

Thus, we can use some encrypted authentication methods to verify the transactions sent from the other chain, such as using Merkle tree, and then analyze the transaction and modify the corresponding state in the chain.

We can create relays on Chain *B*, which would receive the block head of Chain *A* and verify it using the standard verifier of Chain *A*. Once the relay validates the block head, the status of any desired transaction or account can be verified separately by verifying a single branch of the Merkle tree.

Using light client authentication is ideal for relay. In fact, it is impossible for Chain *A* to completely verify Chain *B*, and vice versa. However, through light client verification, Chain *A* contains a small part of Chain *B* and Chain *B* contains a small part of Chain *A* on demand is completely feasible. Suppose a relay on Chain *B* wants to verify a specific transaction or status information on Chain *A*, like a traditional light client, validating the branch of Chain *A*’s encrypted hash tree and then verifying that if the root of the branch is inside the block. If both validations passed, it will consider the transaction or status information to be correct. Because the blockchain is a completely independent environment and cannot access the outside world naturally, the bit information of Chain *A* needs to be transmitted from relay to Chain *B*. However, because the data is cryptography strict self-verification, it is not necessary to trust the relay of the transaction.

The purpose of cross-chain is to enable one chain as a light client of another chain, because we use Byzantine fault-tolerant consistency algorithm [33], light client verification is very simple: all we have to do is check the signature of the latest producer node and verify the Merkle status proof.

Unlike Proof-of-Work consensus algorithm used by Bitcoin and Ethereum, the light client protocol does not need to download and check all the blocks in the block chain. The light client may use the latest block header as long as the producer node has not changed. If the producer node is changing, then the light client needs to track the changes, and in this case, it is necessary to download the block header for each block.

Assuming that we need to communicate across chains, and that we want to send data from Chain *A* to Chain *B*, all we need to do is:

1. Register Chain *A*’s information on Chain *B*, such as chain ID and genesis file configuration information;
2. On Chain *A*, broadcast an outgoing packet destined for Chain *B*;
3. On Chain *A*, broadcast a transaction to Chain *B* to inform Chain *B* of the latest status of Chain *A*, such as block heads and the latest submitted signatures;
4. Send an outgoing packet from Chain *A* to Chain *B*, which includes proof that the transaction has entered the block on Chain *A*. Chain *B* can verify this proof because Chain *B* has the latest block head of Chain *A*.

## 5. Implement of High-Performance Cross-Chain Blockchain Structure

**Figure 3:**
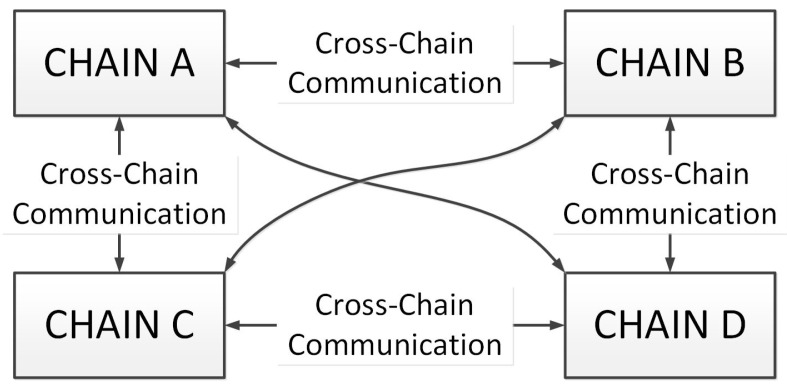
Cross-Chain network structure.

The cross-chain network structure designed in this paper consists of 5 layers from top to bottom: Application Layer, Network Layer, Blockchain Layer, Consensus Layer, and Cross-Chain Layer. An illustration of our cross-chain network is shown in Figure 3.

**Figure 4:**
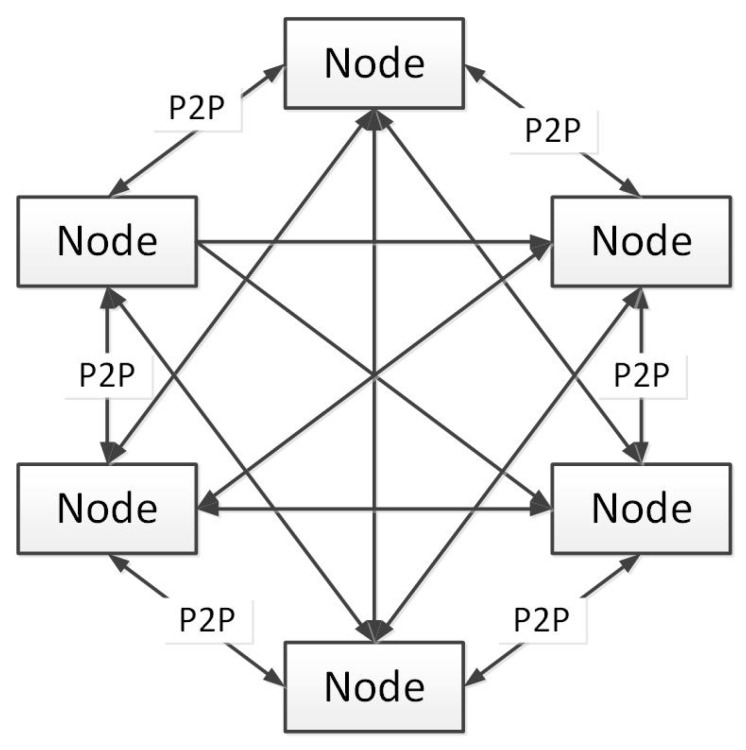
Structure of a single chain.

Each chain in a cross-chain network consists of many nodes. P2P communication network is used between nodes to synchronize the internal state of nodes (as shown Figure 4), reach a consensus with other nodes in the chain, and keep the state of nodes consistent with other nodes. The cross-chain relay between nodes is used to broadcast the transaction between chains.

We will introduce the detail designing of each layer in the following.

### 5.1. Cross-Chain Layer

The designing of the cross-chain layer is the focus of this paper. Relay is explored to synchronize cross-chain transaction data between chains. The source chain (transaction sender) sends the cross-chain transaction to the destination chain. The destination chain, as the light client of the source chain (transaction sender), verifies the effectiveness of the cross-chain transaction, and then changes the status database of the chain to fulfil the cross-chain transaction.

#### 5.1.1. Cross-Chain Transaction Type

Based on the cross-chain protocol introduced in Section 4.2, we have defined 4 kinds of transactions:

1. **ChannelRegisterTx**: The relay node will open a channel first and register its own information to another chain, including the chain ID and the genesis profile of the chain to be registered. This type of transaction occurs only once for a given chain ID and only when two chains need to communicate for the first time. Once the two chains have successfully established communication, subsequent retransmission of this type of transaction will report an exception;
2. **ChannelUpdateTx**: The function of **ChannelUpdateTx** is to update one chain’s state to another chain, which contains the block head and the block signature submitted by the producer node in the chain. Relay node could relay **ChannelUpdateTx**. When relay packets are sent, relay nodes relay these packets to nodes in another chain;
3. **ChannelPacketCreateTx**: The role of this type of transaction is to create outgoing packets on a chain. The packet contains the chain ID of the source and destination chains, the serial number(that is, the integer increments with each message sent between the pairs of chains), the packet type(e.g., Token or data, etc.), and the binary data contained in the packet. Assuming Chain B has an account on Chain *A*, we can use **ChannelPacketCreateTx** on Chain *A* to send funds to that account. We can then prove to Chain *B* that some funds in its account are locked in-chain *A*. These funds can only be unlocked through the corresponding cross-chain transaction message, from Chain *B* to Chain *A* to send the locked funds to that account on Chain *A*;
4. **ChannelPacketUpdateTx**: The **ChannelPacketUpdateTx** is used to release outgoing packets from one chain to another. It contains the data packet and a proof of the status of the packet being submitted to the destination chain. The proof here is the Merkle proof in the tree. We implement a balanced Merklized binary search tree. It contains a list of nodes in the tree that can be hashed together to get the hash of the Merkle root.

#### 5.1.2. Status Storage of Cross-Chain Transaction

We store the status of cross-chain transactions in the Merkle tree for each chain. For each chain that interacts with the current chain, we store the following information: genesis file configuration information, the latest status, block head at the latest block height level, incoming packets, outgoing packets.

The state of the chain is updated each time a block chain transaction is submitted. When a **ChannelPacketCreateTx** transaction is generated, the new packet is added to the exit state. When a **ChannelPacketUpdateTx** transaction is generated, the new packet will be added to the entry state.

#### 5.1.3. Cross-Chain Relay

The relay node is used to handle cross-chain transactions.

For two chains that need to communicate across chains, first, they need to register their own information to each other. The information to be registered includes: the original chain ID, original chain relay node RPC address, the destination chain ID, destination chain genesis file. Registration is accompanied by the construction of a **ChannelRegisterTx** transaction and the booting of the relay node. After the relay node is initialized, the **ChannelRegisterTx** transaction is broadcast to the nodes in the blockchain network.

After completing the relay initialization step, both chains that need to communicate across chains need to open relay nodes to synchronize and transit cross-chain transactions. The relay node polls the queue in the current chain and relays all new queue messages to another chain. At the same time, the relay node will build two types of transactions as needed: **ChannelPacketUpdateTx** to update chain state; **ChannelPacketUpdateTx** to relay transaction data packet to another chain. After building these two types of transactions, the relay node will broadcast them to the nodes in the other chain, waiting for the block nodes to verify and package them into the block.

### 5.2. Consensus Layer

Consensus layer is built based on the contents described in Section 3.

### 5.3. Blockchain Layer

Each block in the blockchain designed in this paper is identified by a hash, which is generated using the SHA256 encryption hash algorithm in the block head. Each block refers to the previous block through the hash field of the previous block in the block head, which is called the parent block. In other words, each block contains its parent hash in its head. Linking each block to its parent hash sequence creates a chain that returns to the first block created, the Genesis block.

The previous block hash field is in the current block’s head, thus affecting the hash of the current block. If the identity of the parent changes, the child’s own identity will change. When the parent is modified in any way, the hash value of the parent changes. The previous block hash field of the child needs to be modified accordingly when the parent hash changes. This causes the child’s hash value to change, and then the need to change the grandson’s pointer, and so on. This cascade effect ensures that once there are multiple generation behind a block, it cannot be changed without forcing recalculations of all subsequent blocks. Because such recalculations require many calculations, the existence of long chain blocks makes the deep history of blockchains immutable, which is the key feature of block chain security.

A block is a data structure of a container type. It aggregates transactions in a blockchain to be included in a public ledger. The block in the chain consists of a block head, followed by a long string of transaction data.

Block head contains the metadata of the block. The data structure of the block head in this paper is shown in Table 1.

#### 5.3.1. Identifier of Block

The main identifier of the block is its encrypted hash, i.e., the digital fingerprint that we generated by hashing the block head using the SHA256 algorithm. This block hash is more exactly the hash of the block head because only the block head is used to calculate it. The block hash uniquely and explicitly identifies the block and may be independently derived by any node by simply hashing the block head.

The second way to identify a block is through its position in the block chain, which is called block height level. Therefore, blocks can be identified in two ways: by reference block hash or by reference block height level. Each back block added to the “top” of the first block is a “higher” position in the blockchain, such as a box stacked on another box.

Unlike block hashes, block height levels are not unique identifiers. Although a single block always has a specific and constant block height level, the block height level does not always identify a single block. When the blockchain splits, two or more blocks may have the same block height level and compete for the same position in the blockchain.

Thus, the block hash of the block always uniquely identifies a single block. Blocks also always have a specific block height level. However, the height level of a particular block cannot always identify a single block. Instead, two or more blocks may compete for a single location in the blockchain.

#### 5.3.2. Merkle Tree

In the blockchain designed in this paper, the Merkle tree is used to aggregate all the transactions in the blocks, generate the overall digital hash of the entire transaction list, thereby providing a very efficient process for verifying whether a transaction is included in the block.

Merkle tree consists of recursive hash node pairs until there is only one hash, which is called the root or Merkle root. The encryption hash algorithm used in our Merkle tree is SHA256. When *N* data elements are hashed and summarized in the Merkle tree, it is possible to check if any one of the data elements is contained in the tree, and only $2\times log_{2}N$ time is required, so it is a very effective data structure.

### 5.4. Network Layer

The blockchain designed in this paper is built on the Internet as a Point-to-Point network architecture. The terms Point-to-Point or p2p means that the computers participating in the network are peer-to-peer, all of which are equal, without a “special” node, and all of the nodes share the burden of network service. The network nodes are topological interconnected in a flat manner in the mesh network. There is no server in the network, no central service, and no hierarchy. A peer-to-peer P2P network simultaneously provides and uses services, reciprocity as a participating incentive. The P2P network has inherent flexibility, dispersibility and openness.

Although the nodes in the P2P network of the blockchains designed in this paper are the same, they may assume different roles in accordance with the functions they support. A node is a collection of following functions: routing, blockchain database, producer and wallet services.

All nodes participate in the routing function of the network, can verify and propagate transactions and blocks, and discover and maintain the connection with point-to-point nodes, or may contain other functions. Only producer nodes maintain the complete and latest copy of the whole blockchain.

When a new node starts, it must find other nodes in the network to participate in the chain. To start this process, the new node must discover at least one existing node in the network and connect to it, which is independent of the geographical location of the other nodes, since the topology of the blockchain network is not defined according to the geographical location. Therefore, any existing node in the chain network can be randomly selected.

The producer node on the blockchain designed in this article maintains a memory pool or transaction pool. It is a list of temporary memory transactions for unconfirmed transactions. The node uses this memory pool to track transactions known in the network but not already included in the block chain.

Some node implementations also maintain a separate isolated transaction pool. If the transaction’s input refers to an unknown transaction, such as a lack of a parent transaction, the isolated transaction is temporarily stored in the isolated transaction pool until the parent transaction arrives.

When a transaction is added to the transaction pool, it is checked if there is an orphan that refers to the output of the transaction (its child). And then verify any matching orphans. If valid, they will be removed from the isolated transaction pool and added to the transaction pool to complete the chain starting with the parent transaction. According to the newly added transaction that no longer is an orphan, the process repeats the search for any other offspring in a recursive fashion until more offspring are found. Through this process, the arrival of the father’s transaction triggered a cascade of reconstructions of a series of interdependent transactions, by realigning the orphans with their parents.

Both transaction pools and isolated transaction pools are stored in node’s local memory. They are dynamically populated from incoming network messages. When the node starts, both pools are empty and gradually populate the new transactions received on the network.

### 5.5. Application Layer

#### 5.5.1. Light Client

We have built the light client tool called crosschaincli. It is a command line tool.

#### 5.5.2. Private Key/Public Key/Address/Account

Public key encryption [34] was born in the 1970s. It is the mathematical basis of modern computer and information security. Since the invention of public key encryption, several suitable mathematical functions have been found, such as prime power [35] and Elliptic curve multiplication [36]. The operation of these mathematical functions is actually irreversible, which means that they are easy to calculate in one direction, but impossible in the opposite direction. Based on these mathematical functions, cryptography can now create digital secrets and unfalsifiable digital signatures.

Elliptic curve multiplication is used as the basis of public key encryption in the blockchain designed in this paper. We use public key encryption to create a key pair that controls access to an asset in a chain. The key pair consists of private key and public key. The public key is used to receive the assets in the block chain and the private key is used to sign the transaction in the block chain. A certain mathematical relationship exists between the public key and the private key. This allows the private key to be used to sign the message, and to verify this message to the public key without the revelation of the private key. When using the asset in the chain, the current asset owner presents its public key and signature in the transaction to spend the owner’s digital asset. By presenting the public key and the signature, each one in the blockchain can verify and accept the transaction as valid, confirming that the person transferring blockchain asset owns them while transferring.

The wallet function of the blockchain node designed in this paper may store the key pair. We use EdDSA [37] method to generate public key from private key. The specific EdDSA method used in this paper is Ed25519, which uses SHA-512 and 25519 elliptic curve. An address is generated from public key through hash.

The account is the status of the user we define in the application layer, in which each account contains a public key, a balance with different token symbols, and a strictly increased serial number.

Accounts are defined to make it easier for users to use, not to represent a user with a hashed public key, i.e., an address, such as Bitcoin and Ethreum. The status of the user changes in the chain as the transaction occurs and is always recorded in the blockchain.

The account is serialized and exists in the Merkle tree with Key as ${}^{\prime }/a/<address{>}^{\prime }$, while <address> is the address of the account. The address of the account is the result of a 20-byte hash of the public key using ${}^{\prime }RIPEMD160^{\prime }$.

#### 5.5.3. Transaction

Transactions are the most important part of the blockchain system, and everything else in the blockchain is designed to ensure that transactions can be created, propagated and validated on the network, and finally added to the global transaction pledger. Transaction is a data structure that encodes the value transfer between users in the blockchain system, in which each transaction is the common data in the blockchain and is shared by the nodes in the whole blockchain network.

In the blockchain designed in this paper, we define a transaction type SendTx, which allows the sending of tokens to other accounts. SendTx receives a set of input and output and sends the token of the corresponding account in the input group to the corresponding account in the output group. The data structure of SendTx is shown in Table 2.

SendFrom in SendTx represents the input of each transaction, with data structure shown in Table 3.

In SendTx, SendTo represents the output of each transaction, with data structure shown in Table 4.

Here Token represents the amount of assets, and the data structure of Token is shown in Table 5.

SendTx is the most basic type of transfer transaction. When a user sends a transaction, the light client builds the transaction and generates SendFrom and SendTo. When the node in the chain acquires the transaction, the validity of the transaction is verified against the signature and public key. If the transaction signature verification fails, the transaction will not continue to be broadcast to other nodes in the blockchain by the node. If the verification is successful, the transaction will continue to be broadcast until the producer node receives the transaction and packs the transaction into block. According to the Inputs of SendTx, producer node will traverse the inputs and outputs, calculate the input and output funds, and calculate the handling fee, and then modify the balance of the input and output summary addresses, and continue to become an irrevocable part of the node state.

Most transactions include transaction fees, which protect the blockchain network to support block generation by producer nodes. The light client will automatically calculate and include transaction fees. Transaction fees are used as an incentive to producer nodes. Transaction fees will be collected by producer nodes as an incentive for producer nodes to package transactions on the blockchain. Transaction fees are calculated based on the size of the transaction, not on the value of the transaction.

Overall, transaction fees are set according to market forces in the network. The producer node determines the priority of the transaction according to many different criteria (including fees), and can even process the transaction free of charge in some cases. Transaction fees affect processing priorities, which means that transactions with sufficient fees may be included in the next block, and for transactions where the transaction fee is too low or there is no transaction fee, the node may delay the processing of these transactions or not even process them.

Transaction fees are not mandatory and can eventually handle fee-free transactions; however, transactions, including transaction fees, give priority to producer nodes to deal with these transactions.

## 6. Experiment and Results

### 6.1. Overview

In the experimental phase, the consensus algorithm designed in this paper will be evaluated and compared with the consensus algorithm of Bitcoin and Ethereum. First, we run the producer node program using docker on each instance, and then we use a separate instance to run the docker container that generates the transaction. The two instances are connected through the internal network, and the purpose of this is to reduce the network delay between the transaction sender and the node program, and to improve the throughput.

The network monitoring tool is used to maintain the active websocket connection to the RPC server of each producer node and to use its local time as the official commit time of the new submitted block the first time it is received.

We first run the experiment without monitoring, by copying all the data in the producer nodes and using the local time of the 2/3 producer nodes of the submitted block as the submission time for the block. The use of monitoring is much faster and is suitable for online monitoring. As long as only block information is passed through websockets, not the entire block, it will be found that it does not affect the result.

The network topology of the nodes is that each producer node is directly connected to each other to reduce the network delay between nodes. For experiments involving crash failure or Byzantine behavior, the number of fault nodes is given by $N_{fault}=\left\lfloor \left(N-1\right)/3\right\rfloor$, where *N* is the total number of producer nodes.

Indicators to be assessed include: (1) Security; (2) High performance.

We will evaluate the security and performance of the cross-chain structure we design. The security refers to the safety of the whole chain, i.e., whether the whole chain can handle the transaction normally in the event of failure of some nodes in the chain, and does not generate the bifurcation behavior. The performance evaluation is the comparison of the efficiency of the transaction processing of the designed cross-chain architecture with the efficiency of the transaction processing of Bitcoin and Ethereum.

We will carry out the experimental analysis on cross-chain network composed of only one single chain or multiple chains on those two aspects.

### 6.2. Experimental Environment

All experiments in this section run on EC2 instances on AWS, using server models t2.medium and c3.8xlarge. t2.medium with 2 vCPU and 4 GB RAM, c3.8xlarge with 32 vCPU and 60 GB RAM. We package the node program of the chain into docker, and run the docker container on each machine. Instances are distributed in seven data centers on five continents to simulate the real environment in which real public chain nodes are distributed around the world.

### 6.3. Single-Chain Evaluation

#### 6.3.1. Security Evaluation

To assess the security effect of this architecture, we manually inject fault nodes to assess the impact on network latency. We set block size as 2048 transactions.

To evaluate the performance and security of the whole chain in the event of an unexpected crash failure, we randomly select and stop $N_{fault}$ producer nodes every three seconds, and restart these stopped producer nodes after three seconds. We vary the value of **TimeoutPropose** to evaluate its influence on transaction efficiency.

Table 6, Table 7, Table 8 and Table 9 show the results of network latency under varied **TimeoutPropose** with different number of producer nodes. As we can see, larger **TimeoutPropose** might help to ease the network latency increase under node failure assumption.

#### 6.3.2. Performance Evaluation

Here, we analyzed the performance of the blockchain designed in this paper under the condition of no external intentional interference, in which all the nodes are online and keep the latest block height level, and there is no Byzantine problem in the blockchain network.

We evaluated for different number of producer nodes from 2 to 64, and the block size varies from 128 to 32,768.

**Figure 5:**
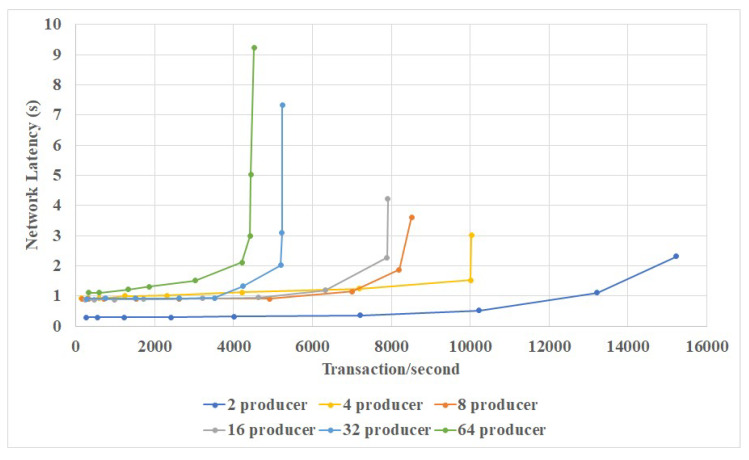
Relationship of network latency and transaction efficiency.

**Figure 6:**
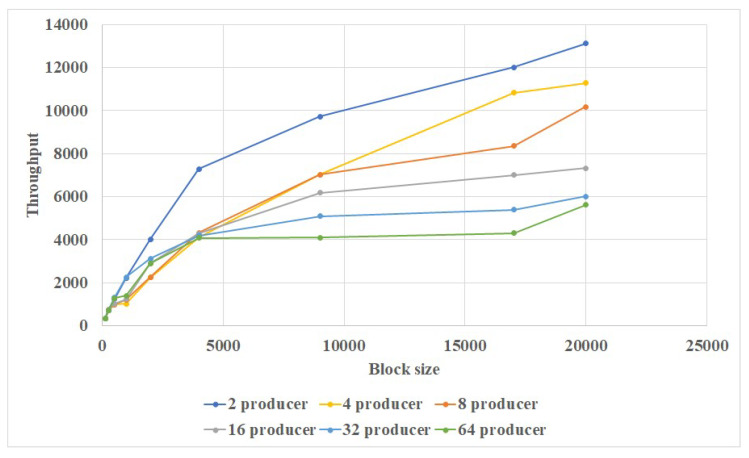
Relationship of throughput and block size.

Results are shown in Figure 5 and Figure 6. It could be seen that our blockchain could easily handle more than 1000 transactions per second. And the network latency could be maintained at 1 s. There is a limit of around 10,000 transactions per second; however, this is much higher than Bitcoin or Ethereum.

The block size of 15,379 transactions has reached 6 MB, and the network bandwidth analysis of producer nodes shows that each connection is easy to reach 20 MB/s. Through the analysis of the log, it is found that when the block size is very large, it may take more than two seconds for the producer node to wait for the blocking part.

**Figure 7:**
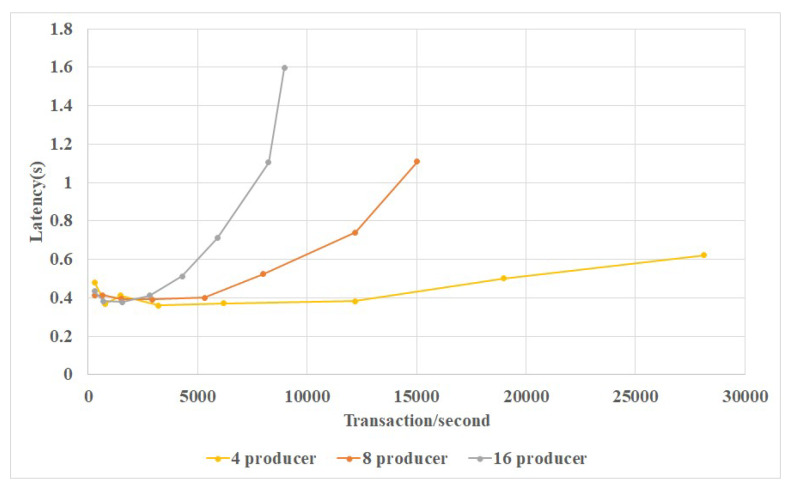
Relationship of transaction amount and block latency for single data center.

Figure 7 shows the performance evaluation result in single data center. It could be seen that network with larger scale could generate higher performance, handle more transaction, produce higher throughput.

**Figure 8:**
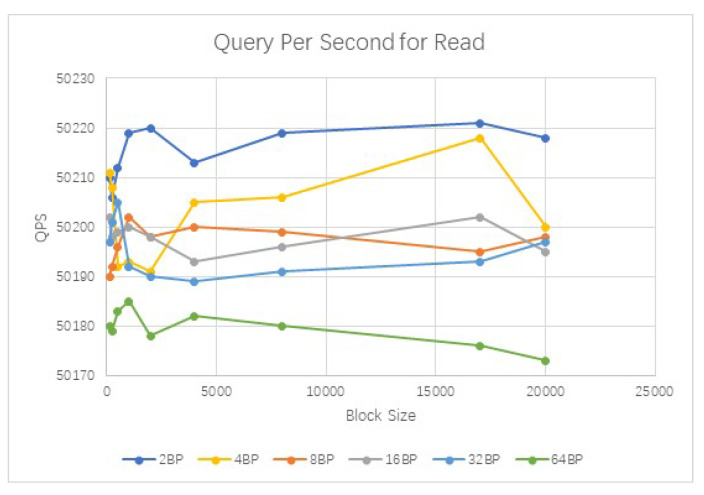
Comparison of the query overhead for reads.

**Figure 9:**
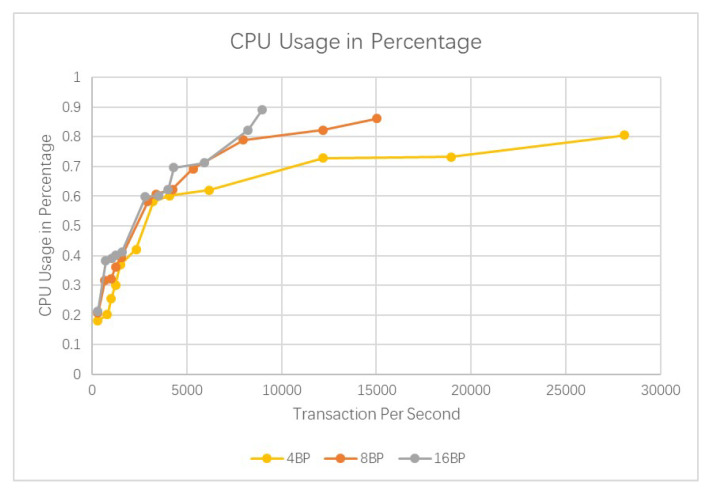
Comparison of the CPU usage in percentage.

**Figure 10:**
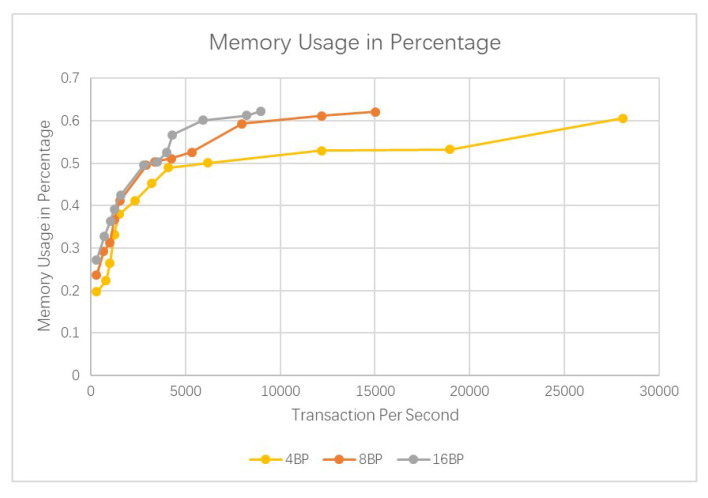
Comparison of the memory usage in percentage.

**Figure 11:**
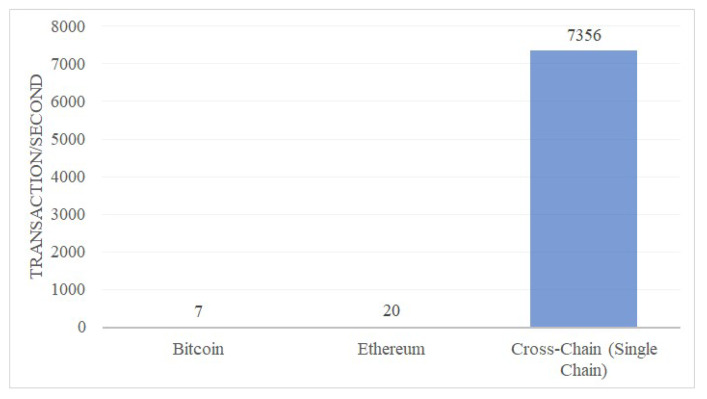
Compare with Bitcoin and Ethereum.

More comparisons are shown in Figure 8, Figure 9 and Figure 10. It could be seen that the average query per second for read is about 50,197. And the CPU usage and memory usage increase with the processed transactions. However, the increasing speed is steady. The comparison with Bitcoin and Ethereum is show in Figure 11. Our cross-chain network has much higher transaction efficiency.

### 6.4. Multiple-Chain Evaluation

A cross-chain network with four chains, namely A, B, C, and D, is built. We evaluate the performance of cross-chain network by calculating the cross-chain transaction efficiency.

**Figure 12:**
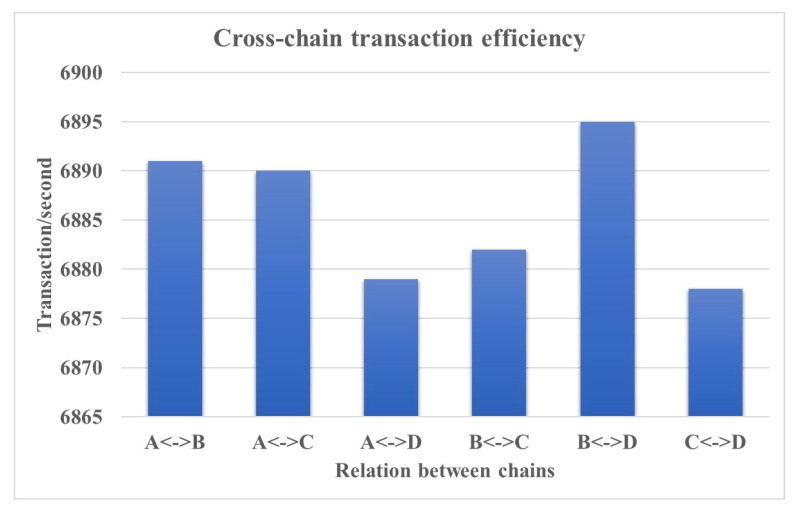
Cross-chain transaction efficiency.

We perform cross-chain transfer between chains, then measure the number of successful transactions been packed into blocks. We set the number of producer nodes as 16, according to the experiment results in Section 6.3. The result is shown in Figure 12. It could be concluded that the cross-chain transaction efficiency is also very high.

## 7. Conclusions

In this work, we have presented a cross-chain network architecture with our improvement on consensus algorithm. By evaluation, we verified this blockchain presented in this paper has higher transaction efficiency, and could handle security issues of blockchain, also verifies our blockchain could be used in real application systems. By implementing the cross-chain, the blockchain designed here could gain higher transaction efficiency, thus is possible to provide support for large-scale business applications.

The summary of comparison with Bitcoin and Ethreum is shown In Table 10. All three architecture could satisfy the requirement of Byzantine fault tolerance. However, Bitcoin and Ethereum do not support cross-chain transaction, also the transaction efficiency is limited. While our blockchain architecture not only could satisfy the security requirement, also has higher transaction efficiency, and implemented based on cross-chain structure, thus could support large-scale distributed applications.

## Figures and Tables

**Table 1:** Structure of block head.

Name	Type	Meaning
ChainID	String	Id of the chain
Height	Int	Height level of the block
Time	Date	Creation time of a block
NumTxs	Int	Number of transactions
LastBlock ID	BlockID	Previous block’s ID
LastCommitHash	[ ]byte	Hash for previous block from producer node
DataHash	[ ]byte	Hash of transaction
BlockProducer Hash	[ ]byte	Hash of the producer node which is packaging the current block
AppHash	[ ]byte	Merkle root of the previous block transaction

**Table 2:** Data structure of SendTx.

Name	Type	Meaning
From	[]SendFrom	Transaction input
To	[]SendTo	Transaction output

**Table 3:** Data structure of SendFrom.

Name	Type	Meaning
From	String	Sender
Token	Token	The amount of token
Sequence	Int	Sequence number
Signature	Signature	Signature
PublicKey	PubKey	Public key of the sender

**Table 4:** Data structure of SendTo.

Name	Type	Meaning
To	String	The transaction receiver
Token	Token	The amount of token

**Table 5:** Data structure of Token.

Name	Type	Meaning
Symbol	String	Symbol
Amount	Int64	Token amount

**Table 6:** Network latency with 4 producer nodes under failure assumption.

TimeoutPropose	Min (ms)	Max (ms)	Average (ms)	Median (ms)	95% Percentile
500	433	15,327	2178	1103	5574
1000	521	18,150	2180	1049	5669
2000	472	15,065	2038	1052	5480
3000	429	9959	2003	1091	5501

**Table 7:** Network latency with 8 producer nodes under failure assumption.

TimeoutPropose	Min (ms)	Max (ms)	Average (ms)	Median (ms)	95% Percentile
500	619	126,320	2701	992	5590
1000	572	9835	1761	963	5837
2000	595	8872	1659	970	5483
3000	536	10102	1635	961	5489

**Table 8:** Network latency with 16 producer nodes under failure assumption.

TimeoutPropose	Min (ms)	Max (ms)	Average (ms)	Median (ms)	95% Percentile
500	783	21,360	1978	1002	5931
1000	759	12,661	1762	983	5645
2000	752	21,283	2043	1006	6871
3000	720	72,403	2392	990	5986

**Table 9:** Network latency with 32 producer nodes under failure assumption.

TimeoutPropose	Min (ms)	Max (ms)	Average (ms)	Median (ms)	95% Percentile
500	761	24,693	2592	1085	14,026
1000	752	19,697	2326	1113	9325
2000	853	21,041	2176	1140	6512
3000	762	25,585	2287	1118	6706

**Table 10:** Comparison with Bitcoin and Ethereum.

Architecture	Byzantine Fault Tolerance	Cross-Chain Transaction	High-Performance
Blockchain designed in this paper	*√*	*√*	*√*
Bitcoin	*√*	×	×
Ethereum	*√*	×	×
